# Role of NADPH-insensitive nitroreductase gene to metronidazole resistance of *Helicobacter pylori* strains

**Published:** 2010

**Authors:** M. Kargar, M. Baghernejad, A Doosti

**Affiliations:** 1 Department of Microbiology, Islamic Azad University, Jahrom Branch, Jahrom; 2 Biotechnology Research Center, Islamic Azad University, Shahrekord Branch, Shahrekord, Iran

**Keywords:** *Helicobacter pylori*, Metronidazole resistance *rdxA* gene

## Abstract

**Background and the purpose of the study:**

Current anti-*H. pylori* therapies are based on the use of two antibiotics with a proton pump inhibitor and/or a bismuth component. Metronidazole is a key component of such combination therapies in Iran. The aim of this study was to determine the role of *rdxA* gene in resistant strains of *H. pylori* isolated from Shahrekord Hajar hospital to metronidazole.

**Methods:**

This study was a cross-sectional method, which was carried out on 263 patients who referred to endoscopy department of Hajar hospital, in 2007. Biopsy samples were cultured on selective *Brucella* agar containing 10% blood and incubated under microerophilic condition at 370C for 3–7 days. Suspected colonies were tested by Gram staining, urease, oxidase and catalase activities. Organisms were confirmed to be *H. pylori* on the basis of the presence of *ureC*(*glmM*) gene by PCR.Specific primers were used for detection of *rdxA* gene mutation.

**Results:**

Eighty and four strains of *H. pylori* determined by PCR method. Of the isolated strains, 49 (58.33%) were resistant, 7 (8.33%) were semi-sensitive to metronidazole and 200bp deletion in *rdxA* gene was observed in 2 strains.

**Conclusion:**

Because of the high metronidazole resistance in patients under study it was necessary to replace it by other antibiotics in therapeutic regimens. On the basis of low frequency of resistance mutation in *rdxA* gene, sequence analysis for identification of other mechanisms is suggested.

## INTRODUCTION

*Helicobacter pylori* is a spiral, gram negative bacterium that has been recognized as a causative factor in gastritis, duodenal and peptic ulcer, gastric adenocarcinoma and MALT lymphoma (1). Resistance of *Helicobacter pylori* to either clarithromycin or metronidazole (Mtz) has been associated with therapeutic failure and reduced eradication rates with multi-agent treatment regimens (2). Multiple nitroreductase are expressed by *H. pylori* and probably contribute to the reductive activation of Mtz (3). In susceptible protozoan and bacterial pathogens to nitroimidazole compounds, reduction of nitro group is considered essential for formation of reductive intermediates which are likely to mediate chromosomal DNA strand breakage with resultant cytotoxity (4). Inactivation mutational of *rdxA* gene, which encoding an oxygen- insensitive (type I) NADPH nitroreductase, confer Mtz resistance on *H. pylori*. It has been considered that *rdxA* gene is the primary nitroreductase responsible for reduction of the nitro group and activation of Mtz in *H. pylori*. Goodwin et al. demonstrated that insertional inactivation of *rdxA* in *H. pylori* resulted in a Mtz-resistance phenotype by preventing reduction of Mtz (3).The aim of this study was to determine the role of *rdxA* gene in metronidazole resistance of *H. pylori* strains isolated from Shahrekord Hajar Hospital in Iran.

## MATERIAL AND METHODS

### 

#### Patients

Totally 263 consecutive patients with dyspeptic symptoms attending the endoscopy suite of gastroenterology section of hospital of shahrekord university of medical sciences from July to December 2007 were enrolled. Each patient's history sheet was examined in detail and findings were recorded on standard performa including demographic data. Those with positive history for above drugs were excluded. All patients read and signed an ‘informed consent’ form at the beginning of endoscopy and declared their willingnesses for the application of their anonymous data for research purpose. For each patient, three biopsy specimens were taken, from the antrum, the gastric body, using a disinfected endoscope, were placed in 0.1 ml of sterile saline solution and sent to clinical microbiology laboratory of islamic azad university in shahrekord.

#### Bacteria and culture conditions

Biopsy samples were cultured on *Brucella* agar (Merck) supplemented with 7% fresh horse blood, vancomycin (6mg/l) (Merck), trimethoprim (5mg/l) (Merck) and amphotricin (2mg/l) (Merck). For praimary culture, plates were incubated at 370C in a microaerophilic atmosphere (5% O2, 15% CO2, 80% N2), for 3–5 days. Strains were identified according to colony morphology,Gram stain and positive reaction with urease, catalase, oxidase.The *ureC (glmM)* which encodes urease was used as a target DNA to confirm *H. pylori* strains.

#### Antimicrobial susceptibility testing

The susceptibilities of the *H. pylori* isolates were examined by an agar dilution method according to CLSI (Clinical and Laboratory Standard Institue) (5). Resistance breakpoint for metronidazole was defined as >8 µg/liter (5).

#### DNA extraction and PCR assays

The extraction of *H. pylori* genomic DNA was performed as reported previously (6). The *ureC* (*glmM*) gene was detected by using the primers 5′-AAGCTTTTAGGGGTGTTAGGGGTTT-3′ and R-5′AAGCTTATTTCTAACGC-3′ with 35 cycle at 39 0C for 1 min, 550C for 1 min, and 720C for 1 min, which amplifies a 295-bp amplicon. PCR reaction was carried out in Gene Amp 9700 (Perkin Elmer) (6). Then seven microliter portions of the PCR products were analyzied by electrophoresis in 1.5% agarose gel using Tris-acetate-EDTA(TEA) buffer stained with ethidium bromide in parallel with a molecular weight marker:Gene ruler 100-bp DNA ladder (MBI Fermentase;Vilnius, Lithuania). For detection of metronidazole resistance, primers RdxA1 (5′-AATTTGAGCATGGGGCGA-3′)and RdxA2 (5′-GAAACGCTTGAAAACACCCT-3’) were used for determination of deletion of *rdxA* gene. PCR amplification was performed in a thermal cycler (PE Applied Biosystems, ChiBA, Japan), as described previously. The sizes of the PCR products of the *rdxA* gene were analyzed by 1.5% agarose gel electrophoresis containing ethidium bromide (0.5ml) (7, 8). The data were analyzed using SPSS software (SPSS for windows, 14 programs) and *Chi-square* and then *Fisher's exact* tests. *P-value* less than 0.05 were taken to indicate statiscal significance.

## RESULTS

### 

#### Culture,RUT and PCR of biopsy specimens

**Figure 1:**
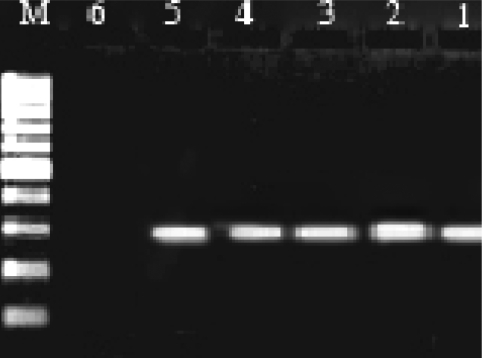
PCR products of *ureC* gene. Lane 1–4 294bp fragments, lane 5 positive control, lane 6 negative control, M; DNA size marker.

*H. pylori* was isolated from 84 of 263 (31.94%) patients participated in this study.Of these 35 (13.31%) were male patients, and 49 (18.63%) were female. The organism was successfully cultured from 55 out of 135 (40.74%) patients with non-ulcer dyspepsia and 29 out of 62 (46.77%) of patients with peptic ulcer. The percentage of culture positive specimens was 31.94 (84 of 263) while a positive RUT and PCR results were observed in 54.37% (143 out of 263), 84.79% (223 out of 263) respectively (Figure 1).

#### Prevalence of metronidazole resistance

**Figure 2:**
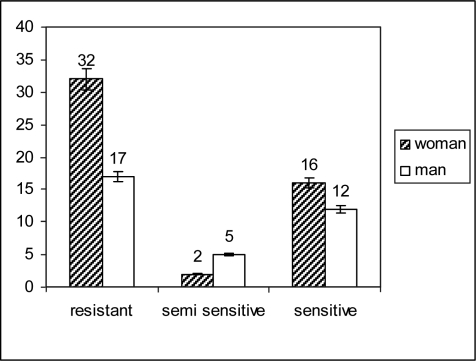
Sensitive, semi sensitive and resistant *H. pylori* strains isolates to metronidazole

**Figure 3:**
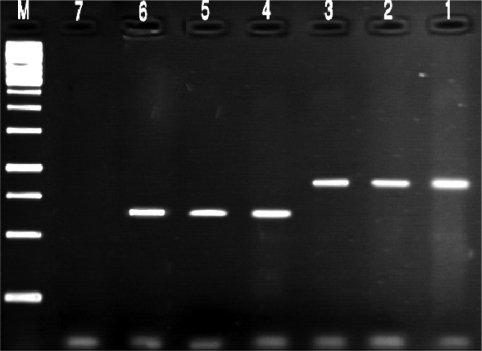
PCR products of metronidazole resistance strains. Lane 1–3 without deletion (800bp), lane 4,5 with 200bp deletion (600bp). Lane 6; positive control, lane 7 negative control, M; DNA size marker (Ladder 100bp).

By an agar dilution method, out of the 84 *H. pylori* isolates, 49 (58.33%) were found to be metronidazole resistant (Figure 2).The results showed no correlation between the metronidazole susceptibilities of *H. pylori* isolates and patients age. There was a significant difference between patient gender and prevalence of metronidazole- resistant *H. pylori*. Out of 49 patients, 17 (20.24%) were male and 32 (38.09%) patients were female harbored resistant strains (*p=*0). For 47 metronidazole resistance strains, the *rdxA* amplicon was approximately 800bp and for 2 resistance strains the *rdxA* amplicon was 600bp (Figure 3). There was no significant difference between *rdxA* gene deletion and Mtz resistance.

## DISCUSSION

A major obstruction to successful *H. pylori* treatment is the presence of antibiotic resistant strains. The prevalence of *H. pylori* resistance to metronidazole varies from 20% to 40% in Europe and USA, with one exception in Northen Italy. It is well known that the prevalence is much higher in developing countries (50–80%), such as Mexico (76.3%) (9, 10). In this study resistance to metronidazole was 58.33%, which was similar to resistance pattern in developing countries. Mtz resistance associated with mutations of *rdxA* gene is still one of the controversial topics. Firstly, Debets-Ossenkopp et al. showed that the 200bp deletion in *rdxA* gene was a major factor in MTZ resistance (8). In contrast, Kato et al. reported that there was no deletion of 200bp in *rdxA* gene in Mtz resistance strains (7). In accordance with these reports, in this study only in 2 (4%) strains, deletion of *rdxA* gene was identified. A troubling aspect of resistance to some antibiotics by *H. pylori* is a phenomenon that has been given the name heteroresistance. Unusually, in testing for resistance, only a single colony of the isolate under study is tested for its susceptibility to various antibiotics. Scientists have now found that if 10 colonies of a strain isolated from an ulcer patient are tested for resistance, they vary widely with respect to susceptibility. This raises the possibility that the appearance of resistance is simply due to selection of the resistant sub-population within the larger population of mostly susceptible bacteria. This phenomenon has been observed so far only with resistance to metronidazole, but it raises the troubling question of how much potential there is for strains of *H.pylori* to become resistant to antibiotics very rapidly (10).

In the present study resistance to metronidazole in women was higher than men, probably due to the use of niroimidazole drugs to treat gynaecological infections. The rate of incidence of *H. pylori* infection in the developed countries may be as low as 30%, while in developing and under developing countries it is more than 80% (11). In this study, 84.79% of patients were infected; this is in agreement with report of Doosti et al. in 2006 in this region (12). However these rates vary wildly in different regions of Iran. For example rates of infection are 62.56%, 65.1% and 48% in Mashhad (13), Isfahan (14) and Semnan (15) respectively.

## CONCLUSION

In summary, mutations in *rdxA* may not always be essential for metronidazole resistance. Future examination of *rdxA* expression at the transcription and translational level may provide further insight into the role of this locus in metronidazole action and resistance of *H. pylori*. On the other hand it seems that other mechanisms such as scavenging of toxic oxygen radicals by an altered catalase or superoxide dismutase is, a more efficient DNA damage repair mechanism, and loss of function of a critical reductase contributed to metronidazole resistance.Thus identification of other resistance mechanisms is suggested.
